# Bioactivity, Cytotoxicity, and Tribological Studies of Nickel-Free Austenitic Stainless Steel Obtained via Powder Metallurgy Route

**DOI:** 10.3390/ma16247637

**Published:** 2023-12-14

**Authors:** Eliza Romanczuk-Ruszuk, Agnieszka Krawczyńska, Andrzej Łukaszewicz, Jerzy Józwik, Arkadiusz Tofil, Zbigniew Oksiuta

**Affiliations:** 1 Institute of Biomedical Engineering, Faculty of Mechanical Engineering, Bialystok University of Technology, Wiejska 45C, 15-351 Bialystok, Poland; 2 Faculty of Materials Science and Engineering, Warsaw University of Technology, Wołoska 141, 02-507 Warsaw, Poland; agnieszka.krawczynska@pw.edu.pl; 3 Institute of Mechanical Engineering, Faculty of Mechanical Engineering, Bialystok University of Technology, Wiejska 45C, 15-351 Bialystok, Poland; a.lukaszewicz@pb.edu.pl; 4 Department of Production Engineering, Faculty of Mechanical Engineering, Lublin University of Technology, Nadbystrzycka 36, 20-618 Lublin, Poland; j.jozwik@pollub.pl; 5 Institute of Technical Sciences and Aviation, University College of Applied Sciences in Chełm, Pocztowa 54, 22-100 Chełm, Poland; atofil@panschelm.edu.pl

**Keywords:** nickel-free austenitic stainless steel, powder metallurgy, wear resistance, bioactivity, cytotoxicity

## Abstract

In the present study, the bioactivity, cytotoxicity, and tribological properties of a nickel-free austenitic stainless steel produced via the mechanical alloying of elemental iron, chromium, and manganese nitride powders following by hot isostatic pressing was investigated. Powders after 90 h of mechanical alloying were consolidated via hot isostatic pressing at 1150 °C (1425 K) and heat treated at 1175 °C (1448 K) for 1 h in a vacuum with furnace cooling. Tribological tests were performed to determine the resistance of the as-received nickel-free steel. It was noticed that applying heat treatment after hot isostatic pressing decreases the average friction coefficient and wear rate of the austenitic steel. An immersion test in a simulated body fluid for 28 days at 37 ± 1 °C has been used to determine the biocompatibility of the tested material. The SEM-EDS analysis allowed us to characterise the morphology of the films and the elements of the steel on the thin-film layer. Elements typical of apatite (calcium and phosphorus) were detected on the surface of the sample. Cellular toxicity tests showed no significant toxic side effects for Saos-2 human osteosarcoma cells and the number of Saos-2 human osteosarcoma cells on the nickel-free steel was greater than on the 316LV grade steel.

## 1. Introduction

Many types of stainless steel grades, e.g., 316L and 316LV, offer good mechanical properties and corrosion resistance, as well as cyto- and biocompatibility. They are the most frequently used implant materials for internal fixation in orthopedics [1]. One of the disadvantages of these steels is release metal ions such as nickel that have been reported to cause allergic reactions in patients [2,3]. The harmful effect of the nickel element on the human body is well known and described in the literature [1,4,5]. Therefore, it is a natural step to develop nickel-free stainless steels as a substitute for conventional stainless steels such as 316L for medical applications [1,5]. Austenitic stainless steels are not only a very popular material used in medical applications, but also in the jewellery and watchmaking industries [6]. It is known that the replacement of toxic and expensive Ni with Mn and N in the steel improves not only the mechanical properties, but also the corrosion resistance and, therefore, the biocompatibility of this type of steels [7]. Additionally, the lower toxicity of steel after the replacement of nickel with manganese results from the greater stability of Mn compounds [8].

Nickel-free austenitic stainless steels comprise one of the most promising materials for implantation, and many scientists and engineers in the field of material science have devoted great efforts to developing new types of austenitic stainless steels without nickel [9,10]. These stainless steels also have good mechanical and corrosion properties and are suitable for various medical devices, such as bone plates, screws, and wires [1,11,12]. Usually, the nickel-free austenitic stainless steel is produced via vacuum induction melting and casting, following by forging at a high temperature (1100–1200 °C) and then long-term annealing and liquid nitrogen quenching. During the melting process, the nitrogen is introduced via the flashing melted method.

Friction is one of the main problems leading to materials being serviced; therefore, it is also important to investigate the wear resistance of nickel-free austenitic stainless steels [13,14]. Thomann et al. [15] have reported that the PANACEA P558 alloy, a high-nitrogen nickel-free austenitic stainless steel, exhibits improved wear resistance compared with 316L [16,17]. Zhao et al. [18] investigated the effect of cold deformation on friction in nickel-free high nitrogen stainless steels in distilled water and Hank’s solution. The results indicated that nickel-free stainless steel has an excellent work-hardening ability, and the dry-wear rate initially decreased then subsequently increased due to increasing cold deformation. Vats et al. [12] studied the tribocorrosion behaviors of nickel-free austenitic stainless steel in simulated body fluids and found that the corrosion potential (Ecor) and the corrosion current density (Icor) values increased with increasing load from 5 N to 10 N.

Yamamoto et al. [19] evaluated the cytotoxicity of an Fe–Cr–Mo–N stainless steel manufactured via nitrogen adsorption treatment and indicated that this steel had higher cell growth than 316L in both static and dynamic conditions. Ren et al. [20] studied the cytotoxicity of a Ni-free stainless steel BIOSSN4 grade and showed its better biocompatibility compared to 316L stainless steel. Fini et al. [4] tested an austenitic nickel-reduced stainless steel named P558, both in vitro on primary osteoblasts and in vivo after bone implantation in a sheep tibia, and demonstrated its good biocompatibility. However, little information is available on the tribological aspect of nickel-free austenitic steel made via the powder metallurgy (PM) route.

Better biocompatibility and wear resistance give nickel-free stainless steel great potential as a biomedical material. In this work, the nickel-free austenitic stainless steel produced via the PM route, including the mechanical alloying (MA) of a mixture of iron, chromium, and manganese nitride powders, and hot isostatic pressing (HIP), followed by heat treatment (HT), was investigated to determine such properties as wear resistance, corrosion resistance, bioactivity, and cytotoxicity. The obtained results were analysed and compared with those for 316LV stainless steel.

## 2. Materials and Methods

### 2.1. Material Preparation

Nickel-free austenitic stainless steel with a nominal composition of Fe-18%Cr-12%Mn-0.5%N was prepared using elemental powders of iron (average particle size ~10 µm, 99.95% purity), chromium (average particle size ~10 µm, 99.92% purity), and manganese nitride (average particle size ~60 µm, 99.95% purity, supplied by Alfa Aesar, Haverill, MA, USA). The MA process was conducted in a stainless steel high-energy planetary ball mill Pulverisette 6 (Fritsch, Idar-Oberstein, Rhineland-Palatinate, Germany), in an argon atmosphere (99.999 purity) with stainless steel balls (10 mm in diameter) and a ball-to-powder ratio (BPR) of 8:1 up to 90 h. The powder after MA was degassed in vacuum, hermetically closed in a low-carbon steel capsule and HIPped under a pressure of 200 MPa, at 1150 °C (1423 K) for 1 h. The material after consolidation was annealed at 1175 °C (1448 K) for 1 h in a vacuum with furnace cooling (denoted as HIP + HT). More details of the mechanical alloying and consolidation process are presented in the literature [21].

For microstructure observations, the samples were mechanically ground and polished, etched, and analysed using Scanning Electron Microscope (SEM, Hitachi 3000N, Tokyo, Japan), Transmission Electron Microscope (TEM, Jeol JEM 1200, Tokyo, Japan) and Scanning Transmission Electron Microscopy (STEM, Hitachi STEM HD2700). The chemical composition and density of the tested materials were presented in our previous work [20].

### 2.2. Tribology Testing

**Figure 1:**
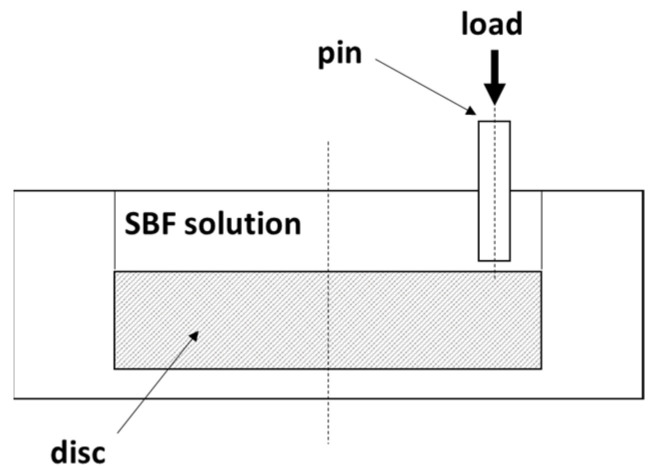
Schematic view of a pin-on-disc configuration.

Tribological studies were carried out using a UMT TriboLab tribometer (Bruker, Billerica, MA, USA), with a pin-on-disc system in a simulated body fluid (SBF) solution (Figure 1). The duration of each measurement was 3600 s at room temperature (RT). Each measurement was repeated three times. Abrasion resistance was tested with the following parameters: normal force (Fn) = 5 N, frequency (f) = 1 Hz. The tested material was used for the pin. The counter sample was a 316LV stainless steel disc.

The friction coefficient was continuously recorded using a load cell. The wear of the nickel-free stainless steel pin was estimated using the specific wear coefficient (Ka), given by:

(1) $$K_{a}=\frac{V}{F_{n}S}$$

where V [m^3^] is the measured wear volume calculated using the bulk density; Fn [N] is the applied load; and S [m] is the sliding distance.

The disc was made of 316LV stainless steel. The wear rate (WR) was assessed via weighting the test sample before and after each run with an analytical balance with a 10^−4^ g sensitivity and calculated using Formula (2):

(2) $$WR=\frac{\left(Mass\;of\;pin\;before\;wear\;test-mass\;of\;pin\;after\;wear\;test\right)}{Sliding\;distance}$$

### 2.3. Bioactivity Analysis

The in vitro bioactivity of the HIP and HT samples was studied via soaking the specimens in the SBF. The samples, with a size of Ø 8 × 4 mm, were prepared. The samples were polished with rough sandpaper (800 grid), ultrasonically rinsed in acetone, and dried. SBF solution was prepared through the dissolving in 1 L of deionised water of a reagent composed of sodium chloride (NaCl), potassium chloride (KCl), calcium chloride dihydrate (CaCl_2_·2H_2_O), magnesium chloride hexahydrate (MgCl_2_·6H_2_O), sodium hydrogen carbonate (NaHCO_3_), dipotassium hydrogen phosphate trihydrate (K_2_HPO_4_·3H_2_O), and sodium sulphate (Na_2_SO_4_). Then, the solution was buffered to physiological pH 7.4 at 37 °C (310 K) by both hydrochloric acid (HCl) and tris (hydroxymethyl)-aminomethane ((CH_2_OH)_3_CNH_2_) [21]. The pH value of SBF, as obtained, was similar to that of human blood plasma, as shown in Table 1. The tests were performed at 37 °C (310 K) for 7, 14, 21, and 28 days under dynamic conditions (the solution was changed every 7 days).

The tested samples were placed in sealed vessels containing 25 cm^3^ of the SBF solution. The volume of the solution (Vs) in cm^3^ was determined using Formula (3) [22]:

(3) $$V_{s}=\frac{S_{a}}{10}$$

where Sa is the surface of the sample [mm^2^].

After immersion in the SBF for various periods, the samples were retrieved, gently rinsed with distilled water, and dried. The surface of the samples was examined using SEM (Hitachi 3000N) and XRD (D8 Eco Advanced, Bruker, Billerica, MA, USA). The pH of the solution was tested after 7, 14, 21, and 28 days.

### 2.4. Cytotoxicity Testing

For the cytotoxicity study, the Saos-2 human osteosarcoma cell line (ATCC^®^ HTB-85TM) (ATCC-American Type Culture Collection, Manassas, VA, USA) was used. Before the test, the samples were mechanically ground and polished, then washed in demineralised water in an ultrasonic cleaner for 900 s, and sterilised in an autoclave at 121 °C (394 K) for 0.5 h. Cytotoxicity tests of all specimens followed International Standards ISO 10993-5 After reaching 75% confluency, cells were harvested and incubated with the extracts for 48 h at 37 °C (310 K) and 5% CO_2_ environment. Then, the cells were seeded on samples with the density of 6 × 10^4^ cells/well and were cultured under 37 °C (310 K), 5% CO_2_ environment for 48 h. Positive and negative controls were also used to confirm the good performance of the assay. Cell viability was measured via XTT assay.

### 2.5. Corrosion Testing

Corrosion resistance was measured according to International Standards ISO 10993-15. The PGP201 VoltaLab galvanostat/potentiostat (Radiometer Analytical, Lyon, France) equipped with the VoltaMaster 4 software was used. The corrosion medium was 70 mL of Hank’s solution. More details on the study are described in [20]. The following corrosion parameters are given here: polarization resistance (Rp), corrosion current density (Icor), corrosion potential (Ecor), and corrosion rate (CR).

### 2.6. Statistical Analysis

Data obtained in this study were statistically analysed using the statistical software Statistica 13, and reported as means ± standard deviation. To reveal differences among the tested material groups, a one-way ANOVA followed by a test was used. Differences were considered significant at *p* < 0.05.

## 3. Results

### 3.1. Microstructural Characterisation

**Figure 2:**
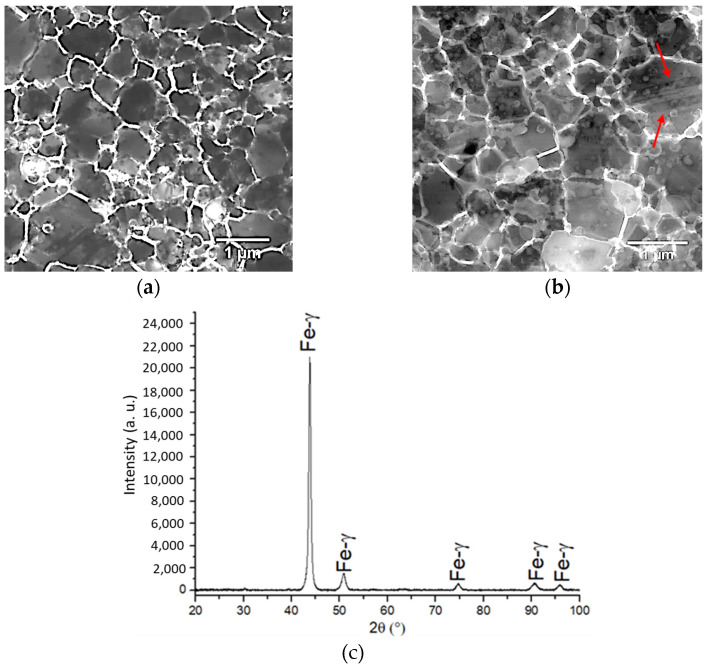
TEM micrographs of the (**a**) as-HIPed material and (**b**) HIP + HT (red arrows indicate tweens), and (**c**) XRD data of the HIP + HT sample.

The density of the Fe-18Cr-12Mn-0.5N (wt. %) stainless steel bulk composition after HIP + HT was 96.8 ± 0.24% of the theoretical density of the austenitic stainless steel (calculated theoretical density of the steel ρ = 7785 kg/m^3^). The heat treatment did not significantly affect the density of the as-HIP steel (less than 1%); however, the nitrogen content in the HIP + HT material decreased about 10%, compared to the HIP material. The other elements in all the tested samples were in nominal amounts (18 ± 0.20% of Cr, 12 ± 0.15% of Mn). Figure 2a,b show TEM images of microstructure of the as-HIP and HIP + HT of nickel-free steel samples. The HIP + HT microstructure consists of the homogeneous and small grain size 2.47 ± 0.49 μm, as well as annealing twins (red arrow in Figure 2b). The XRD analysis confirmed a fully austenitic phase structure, as presented in Figure 2c [21].

In the material obtained after etching, relatively clear grain boundaries are visible (image analysis). Compared to the work presented by Heidari L. et al., in which the hot powder forging and binder-assisted extrusion methods were used, sintering aid porosity and precipitations were observed after etching [23]. The grain size in the material obtained in this paper is smaller compared to the nickel-free austenitic stainless steel presented by Chao et al. [24]. However, in the research work by Chao et al., [24] the method of melting in an argon and nitrogen mixture was used to obtain nickel-free stainless steel, and then the obtained material was treated at 1180 °C (1453 K), hot-rolled, and cooled to room temperature in air. Despite the different methods of stainless steel preparation, the advantages of the PM manufacturing route in terms of the microstructure refining of the steel can be emphasised.

**Figure 3:**
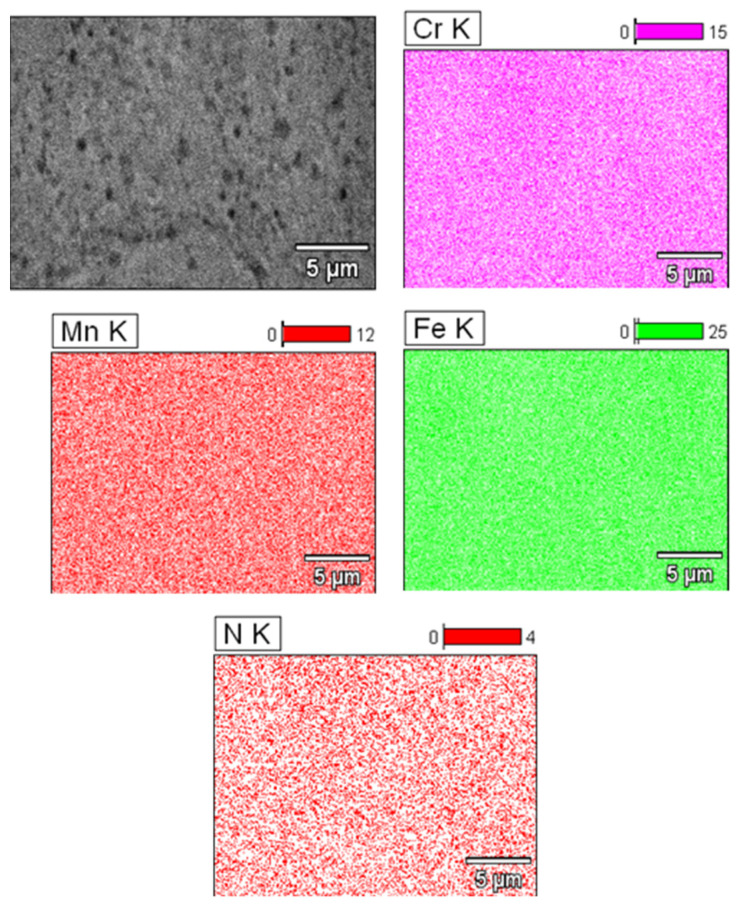
SEM-EDS analysis of the HIP material.

Figure 3 shows a SEM-EDS image of the distribution of alloying elements. The analysis shows that the alloy deposits are regularly distributed. This proves that the powders were alloyed during the mechanical alloying process, and then sintering using the HIP method allowed us to obtain a material with a density above 95%.

#### Cytotoxicity Tests

**Figure 4:**
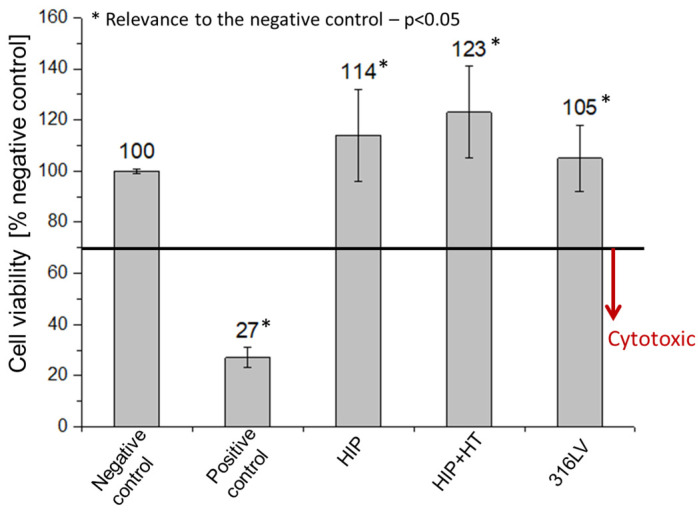
XTT assay of the cytotoxicity of the produced Fe-18%Cr-12%Mn-0.5%N nickel-free materials after HIP and HIP + HT compared to 316LV stainless steel.

ATCC^®^ HTB-85TM (human osteosarcoma cell line) was cultured on the nickel-free stainless steel samples obtained via HIP and HIP + HT and, for comparison, on 316LV stainless steel. The cell proliferation was assessed after 48 h for all the tested materials. The cell viability was assessed using cell viability graph analysis, and the results were compared with the control. Figure 4 shows the comparative results, indicating that proliferation increased in all materials tested compared to the control. The HIP + HT sample showed the highest cell proliferation, indicating the highest antibacterial properties among all the tested materials. These results indicate that all tested materials are non-cytotoxic, in accordance with the International Standards EN ISO 10993-5, and the cell viability did not fall below 100% of the negative control value.

### 3.2. Bioactivity and Corrosion Tests

**Figure 5:**
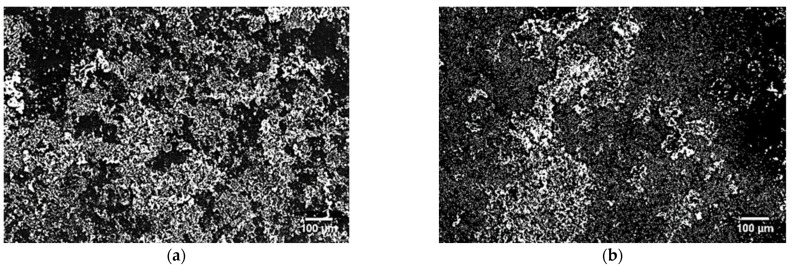
SEM images of the surface morphology of the HIP + HT samples after immersion for 7 days in the SBF solution: (**a**) homogeneous distribution, (**b**) an area with a lower apatite concentration.

The SEM morphology of the samples surface after 7 days of immersion in the SBF solution is shown in Figure 5. It was observed that the apatite had grown on the surface of all tested samples. However, on the surface of the HIP + HT nickel-free stainless steel, the apatite is visible on the entire surface (Figure 5a), while areas with a lower apatite concentration were detected on the surface of the HIP stainless steel, as shown in Figure 5b. The irregular distribution of appetite on the surface of the tested materials indicates the gradual deposition of hydroxyapatite (HA) on the surface of stainless steel.

**Figure 6:**
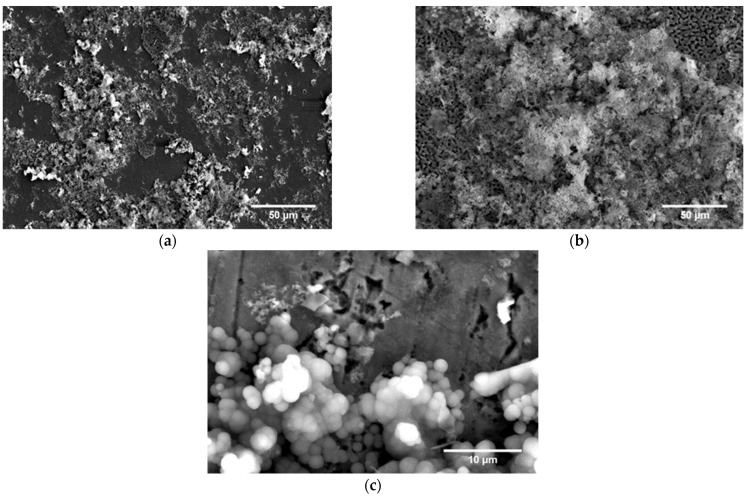
SEM image of surface morphology after immersion for 28 days in SBF solution: (**a**) HIP, (**b**,**c**) HIP + HT.

In each tested material, the apatite on the surface increased after 28 days of exposure (see Figure 6). SEM images of HIP and HIP + HT materials after 7 and 28 days of incubation indicate a gradual increase in hydroxyapatite on their surface. Initially, small apatite nuclei form on the surface of the sample, due to porosity or surface roughness, that increase the surface energy, and then expand to cover larger areas. It is worth noting that in the HIP + HT material, after 28 days of incubation, apatite appears in two different forms of morphology: as a fine dendritic structure with a strongly developed surface area and in a globular (spherical) form.

**Figure 7:**
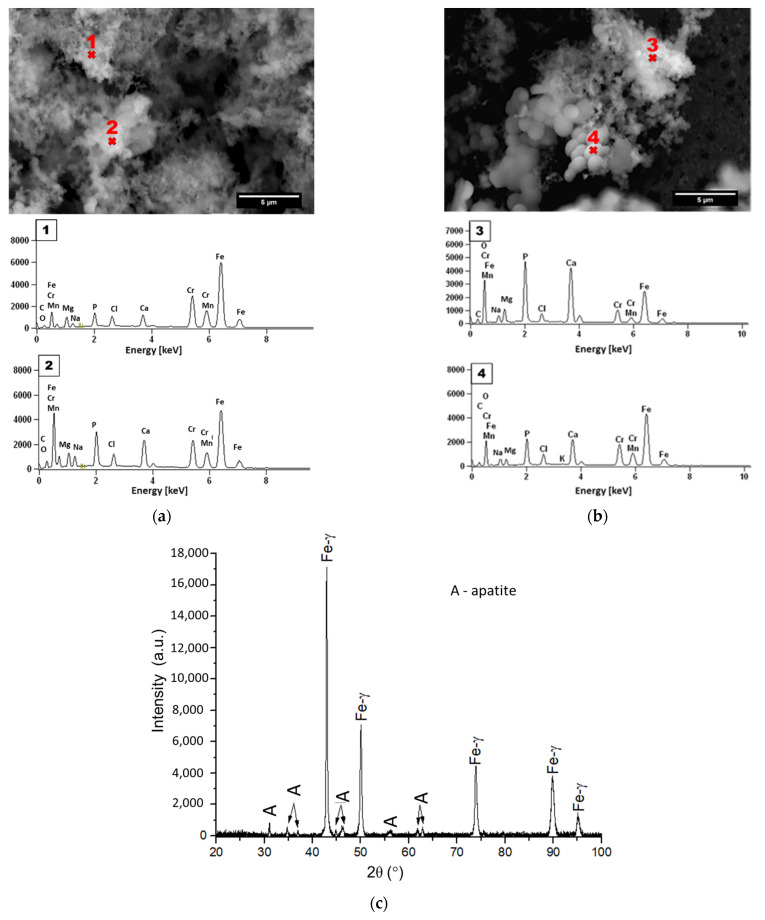
The SEM-EDS spectra of the surface of HIP + HT sample after immersion in SBF for (**a**) 7 days and (**b**) 28 days, and (**c**) the XRD of the HIP + HT sample after 28 days of incubation (A—apatite).

In order to confirm that the layer formed on the materials is hydroxyapatite, the chemical composition of the sample surface was analysed using the SEM-EDS method (Figure 7a,b, Table 2). The SEM-EDS spectra show a composition of elements typical to apatite (including Ca, P with the Ca/P ratio of ~1.70). It confirms the formation of the HA layer on the surface of the tested sample. In addition to the SEM-EDS observations, XRD analysis confirmed the formation of a calcium-rich structure, presented in Figure 7c.

**Figure 8:**
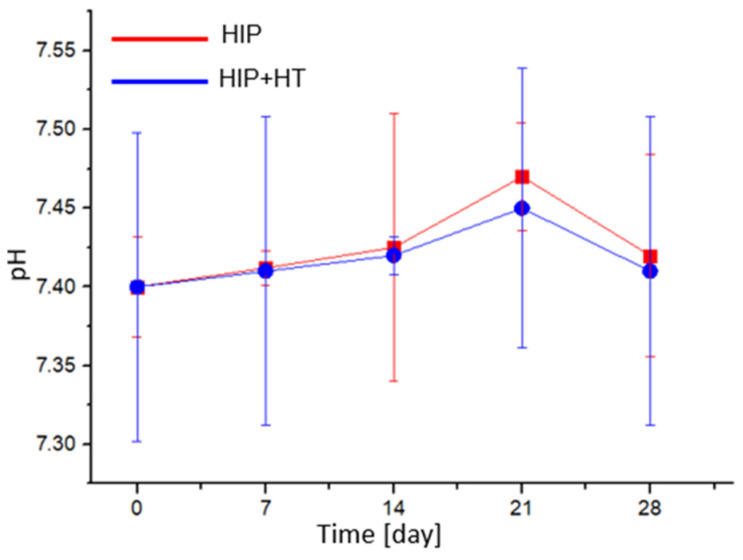
Changes in the pH of the SBF solution at different conditioning times for the tested samples.

Tissue fluids circulate constantly in the human body, so it can be assumed that a more appropriate method for testing bioactivity is the approach with regular replacement of the SBF solution. In Figure 8, it can be seen that the changes in the pH of the solutions of all tested materials are similar; the pH increases up to 21 days of incubation, and then decreases. It may be related to phosphate groups and calcium ions being adsorbed and deposited on the steel surface in the form of calcium-phosphate compounds. This stage of apatite formation lowers the pH in the SBF solution [25].

Table 3 shows the corrosion parameters of the tested materials and 316LV stainless steel. The corrosion potential is highest in the HIP + HT material, while the polarisation resistance is the highest for 316LV steel. Additionally, the corrosion rate of the HIP + HT material is similar to that of 316LV steel, while the material after HIP has the highest corrosion rate. It can be noted that the heat treatment after HIP significantly affects the corrosion properties. The corrosion resistance of PM steels is related to the density and pore morphology of the HIP and HIP + HT samples [21].

Comparing the presented results with the literature data, it can be concluded that the material obtained in this work has very promising corrosion resistance. Yu et al. [26], investigating the effect of nitrogen content on the corrosion properties of stainless steel, showed that a higher nitrogen content improves corrosion resistance. In the material with the chemical composition Fe-17Cr-11Mn-3-Mo-1.2N (in wt. %), the corrosion potential was −0.469 V, which is a similar value to the material obtained with the HIP method in this work. The corrosion potential (Ecor) and corrosion current density (Icor) of the HIP + HT material were higher and lower, respectively, compared to the Fe-18Cr-15Mn-0.66N (in wt. %) steel tested by Qiao et al. [27]. It is worth noting that in work [27], the corrosion tests were carried out in a 3.5% NaCl solution. In this study, SBF solution was used, which has more ions that affect the corrosion process. In the work of [28], the corrosion resistance of the Fe-17Cr-10Mn-4Mo-0.4Si-0.4Fe3N-0.2C (in wt. %) steel prepared via hot forging was investigated, which turned out to be lower compared to the results presented in this work.

### 3.3. Tribology Testing

**Figure 9:**
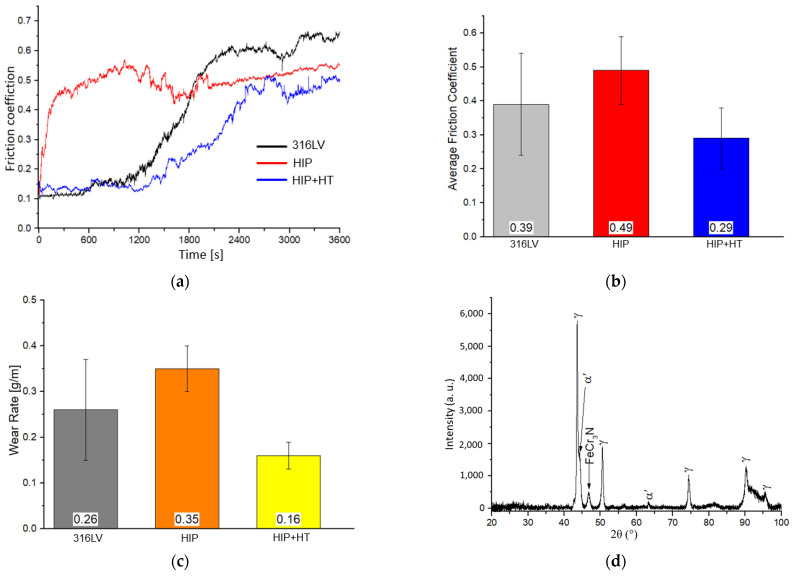
(**a**) Plots of the friction coefficient of the tested materials vs. the time; (**b**) the average friction coefficient; (**c**) the wear rate of the tested materials; (**d**) the XRD pattern of the HIP + HT material after friction tests.

The friction coefficient (FC) vs. the time of the process presented in Figure 9a shows that both HIP and HIP + HT samples have lower values of this parameter after 3600 s than 316LV steel. The average FC value and wear rate from the experiments are shown in Figure 9b and Figure 9c, respectively. The average FC coefficient for the different materials tested obtained different values, ranging from 0.29 to 0.49. The friction coefficient of the HIP material initially increases rapidly and stabilises after approximately 300 s, then gradually increases after 1200 s and stabilises at the value of 0.5 after 1800 s. A completely different progression of friction coefficient changes applies to 316LV and HIP + HT materials. In these materials, FC is initially stable, then, after 1200 s, it begins to gradually increase, faster for 316LV steel. It is worth noting that the FC of 316LV steel after 3600 s of testing reached the highest value (~0.65), but the highest average value of this parameter was calculated for the HIP sample. The lowest wear rate was measured in the HIP + HT sample and it was characterised by the lowest average coefficient of friction (Figure 9c). The lowest coefficient of friction and wear rate give the highest wear resistance during the surface abrasion mechanism. As expected, the lower-density HIP sample has a lower friction coefficient. Note that HIP + HT material has also the lowest microhardness of 300 HV_0.2_, while the HIP sample has the approximately 45% higher hardness value of 435 HV_0.2_.

Figure 9d shows the X-ray diffractogram of the HIP + HT material after tribological testing. It should be noted that after testing, martensite (α’) is observed on the surface of the HIP + HT sample, probably due to the strain-induced martensitic transformation of austenitic stainless steels caused by excessive plastic deformation [29]. Interestingly, this transformation was not observed for the as-HIP sample. The γ to α’ phase transformation may be responsible for the lowest values for the friction coefficient and wear rate of the HIP + HT sample, as it gradually strengthened the surface of the material during friction. Also, complex and hard FeCr_3_N precipitations, detected via XRD, which were formed during the friction process as a result of the reaction of nitrogen dissolved in the steel matrix with chromium and iron, can improve the tribological properties of the HIP + HT steel sample.

Small changes in the friction coefficient of the HIP sample during the test may be caused by the contact instability when the indenter ploughed against the samples [29,30]. Qiao et al. [14] reported that, for nickel-free stainless steel Fe-19Cr-15Mn-0.66N (in wt. %), the average friction coefficient at a load of 5 N was 0.513, and was higher compared to the materials tested here. These differences are probably due to the use of a different friction-testing method.

## 4. Discussion

The newly developed high-nitrogen nickel-free stainless steel with Fe-18%Cr-12%Mn-0.5%N is a reliable substitute for the conventional medical 316LV stainless steels. According to International Standards ISO 5832-1, the stainless steel used for implants should have a single-phase austenitic microstructure, with a fine grain size, and small nitride and carbide precipitation, to ensure a good combination of mechanical and corrosion properties. It is known that nitrogen is a strong stabiliser of austenite and, together with Mn, can replace toxic nickel in steels [9,31]. As a result, it is possible to obtain nickel-free stainless steel with better properties than steel with the addition of nickel.

The changes in the pH of the SBF solution observed during the bioactivity tests of the HIP + HT nickel-free stainless steel may be caused by the adsorption of Ca^2+^ and PO_4_^3−^ ions (Figure 8). Ca^2+^ and PO_4_^3−^ ions, after being deposited on the material surface, enhance nucleation and gradual crystallisation in apatite. The ability to form apatite depends on many factors, one of which is the presence of phosphate groups in the solution, which occur mainly at a pH above 7.2 [22].

Based on the observed results of bioactivity studies and the literature on the subject, a mechanism for the growth of the apatite layer on a nickel-free stainless steel surface is proposed here. In an environment with OH^−^ ions, the first stage is the reaction taking place at the steel–solution interface, which involves binding OH^−^ ions and forming a thin layer of Cr(OH)_3_. This phenomenon is explained by the lower free energy of the formation of the Cr(OH)_3_ compound compared to the Cr_2_O_3_ oxide [5,32,33]. Chromium (III) hydroxide covers the steel surface, and some of this compound is further combined with chromium atoms from the tested material and with OH^−^ ions from the solution, transforming into chromium (III) oxide (Cr_2_O_3_), according to the reaction (4):

Cr(OH)_3_ + Cr + 3OH^−^ → Cr_2_O_3_ + 3H_2_O + 3e^−^ (4)

A layer of chromium oxide (Cr_2_O_3_) is then formed on the sample surface. This layer is porous and has structural defects, which leads to further chemical reactions. The chromium oxide layer reacts with Na^+^ and OH^−^ ions in the SBF solution, and the following reaction takes place on the surface of the material (5):

8Na^2+^ + 8OH^−^ + Cr_2_O_3_ → Na_4_CrO_4_ + H_2_O + H_2_ (5)

**Figure 10:**
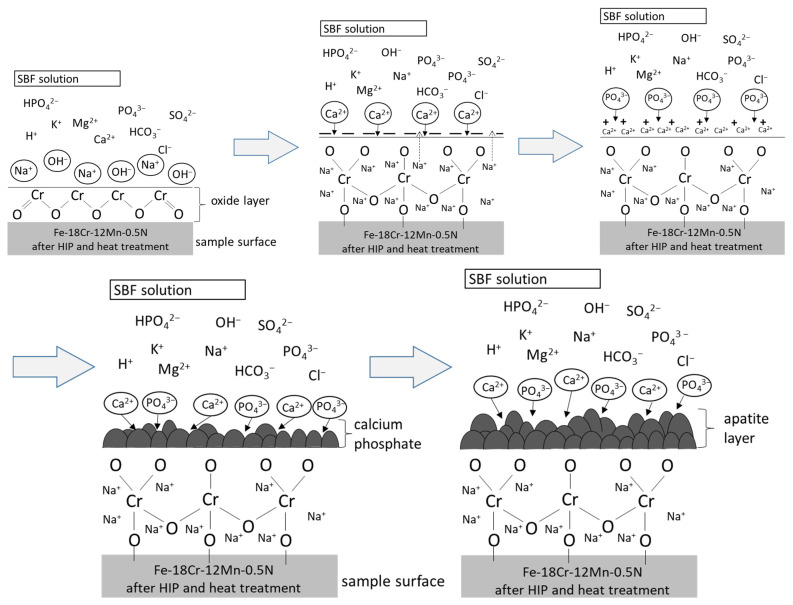
A schematic view of the mechanism of the apatite layer formation on the surface of the HIP + HT sample.

Figure 10 shows a schematic view of the apatite layer formation described above.

As tribological tests of the tested materials showed that in the initial stage of friction in the SBF solution, the friction pair was pressed into the sample surface under a normal load, the contact area was abnormally small, and the macroscopic contact stress was very large. When the friction pair moved, it ploughed the wear surface with high friction resistance, resulting in a high friction coefficient. As the wear progressed, the contact area of the friction pair with the wear surface gradually increased and the macroscopic contact stress decreased, so, after the initial sharp increase, the friction coefficient stabilised [14,18]. The lowest FC value and highest wear resistance were measured for the HIP + HT material, probably due to the hard nitride complex precipitations detected via XRD, despite the initial lowest hardness of this sample in comparison to the HIP conditions. A characteristic feature of the HIP + HT and 316LV materials, contrary to the HIP sample, is that in the first stage of wear, contact surfaces are lapped, and a transfer layer is formed, as confirmed by a slight increase in the mass of the pin (Figure 9a), and the material loss is negligible. After this initial period that takes about 1200 s, the increase in temperature causes the increased number of joints that cause wear to transition as new wear mechanisms emerge. The mechanical strength obtained as a result of heat treatment provides the load-bearing capacity necessary to avoid extensive plastic deformations in the surface layers and, consequently, shear phenomena and the γ to α’ phase transformation. As the wear test progresses, the local temperature increases, and at a certain temperature, appropriate conditions are reached to promote the oxidation of the steel layer. Thus, a wear transition rapidly occurs, and the degree of wear increases significantly.

## 5. Conclusions

The bioactivity, cytotoxicity, corrosion, and wear resistance of the nickel-free austenitic stainless steel prepared via the powder metallurgy route were compared with AISI 316LV steel. The following conclusions can be drawn:

1. Nickel-free stainless steel Fe-18%Cr-12%Ni-0.5%N obtained from elemental powders using MA, HIP, and HT is characterised by a small grain size and a fully austenitic phase structure with annealing twins.
2. Vacuum heat treatment of nickel-free steel applied after HIP reduces, on one hand, the hardness, and improves, on the other hand, the corrosion, wear resistance and cytotoxicity of the HIP nickel-free stainless steel.
3. In vitro cytotoxicity studies indicate that none of the tested materials showed any cytotoxic effect. However, in the case of the HIP + HT sample, an increased cell proliferation was observed.
4. During bioactivity tests, an apatite layer appeared on all tested materials after 7 days of immersion in the SBF solution.
5. Friction tests of the tested materials revealed that HIP + HT nickel-free stainless steel has the lowest friction coefficient and wear rate, even better than 316LV steel.

## Figures and Tables

**Table 1:** Chemical composition of the simulated body fluid solution (SBF).

Reagent	Amount
NaCl	8.035 g
NaHCO_3_	0.355 g
KCl	0.225 g
K_2_HPO_4_∙3H_2_O	0.231 g
MgCl_2_∙6H_2_O	0.311 g
1.0 M HCl	39 cm^3^
CaCl_2_	0.292 g
Na_2_SO_4_	0.072 g
Tris—(CH_2_OH)_3_CNH_2_	6.118 g
1.0 M HCl	Appropriate amount for adjusting pH

**Table 2:** SEM-EDS point analysis of chemical composition of the surface of HIP + HT material after 28 days of incubation.

Element	Chemical Composition [% mas.]				
	Point 1	Point 2	Point 3	Point 4	Average
Ca	24.18 ± 0.08	19.56 ± 0.13	20.61 ± 0.11	23.16 ± 0.17	21.88 ± 0.11
P	15.05 ± 0.14	11.18 ± 0.09	12.01 ± 0.07	12.98 ± 0.12	12.81 ± 0.09

**Table 3:** Summarised corrosion properties of the tested specimens and 316LV stainless steel.

Material	Ecor (V)	Rp (ohm)	Icor (µA/cm^2^)	CR (µm/cm^2^)
HIP	−0.495 ± 0.023	6740 ± 155	2.090 ± 0.012	147 ± 5
HIP + HT	−0.072 ± 0.026	473,790 ± 1236	0.023 ± 0.004	0.913 ± 0.07
316LV	−0.140 ± 0.012	452,300 ± 1450	0.030 ± 0.003	0.873 ± 0.09

## Data Availability

Data are contained within the article.
